# Telemedicine in Times of the Pandemic Produced by COVID-19: Implementation of a Teleconsultation Protocol in a Hospital Emergency Department

**DOI:** 10.3390/healthcare8040357

**Published:** 2020-09-23

**Authors:** Antonio Lopez-Villegas, Salvador Maroto-Martin, Miguel Angel Baena-Lopez, Antonio Garzon-Miralles, Rafael Jesús Bautista-Mesa, Salvador Peiro, Cesar Leal-Costa

**Affiliations:** 1 Social Involvement of Critical and Emergency Medicine, CTS-609 Research Group, Hospital de Poniente, 04700 Almeria, Spain; antonio.lopez@ephpo.es; 2 Emergency Service Hospital, Hospital de Poniente, 04700 Almería, Spain; salvador.maroto@ephpo.es; 3 Deputy Medical, Hospital de Poniente, 04700 Almeria, Spain; miguelangel.baena@ephpo.es; 4 Computer Systems and Communications Area, Hospital de Poniente, 04700 Almería, Spain; antonio.garzon@ephpo.es; 5 Management Unit, Hospital de Poniente, 04700 Almeria, Spain; investigacion@ephpo.es; 6 Health Services Research Unit, FISABIO-PUBLIC HEALTH, 46020 Valencia, Spain; peiro_bor@gva.es; 7 Nursing Department, University of Murcia, 30100 Murcia, Spain

**Keywords:** COVID-19 disease, health professional security, teleconsultation, telemedicine, emergencies

## Abstract

Since the first case of COVID-19 was reported in Spain, almost 22% of healthcare professionals have been infected. Among the main causes are exposure during the care of suspected patients and asymptomatic patients, which caused a greater lack of protection in some cases, and to the global shortage of personal protective equipment due to the strong demand for it. The main objective of this study was to evaluate the effectiveness of a teleconsultation protocol with patients who had respiratory symptoms in the reduction of the consumption of personal protective equipment (PPE) in a hospital emergency service (HES) during the COVID-19 pandemic. This is a descriptive and retrospective study that analyzes the implementation of a teleconsultation protocol with patients with respiratory problems treated in the HES at the Hospital de Poniente (Almeria), between 18 March and 30 April 2020. In the selected study period, 5353 patients were treated in the HES of the Hospital de Poniente; of these, 15.43% showed respiratory symptoms and were referred to the Respiratory Circuit, of which 42.2% did so via teleconsultation. Sixty-six cases of COVID-19 were diagnosed, 57.6% were male, and the median age was 71 years old. The main disease related was pneumonia (89.4%), symptoms more frequent were cough (77.3%), fever (77.3%), and dyspnea (60.6%). Lastly, 56.1% of the patients that attended had one or more comorbidities, high blood pressure (53%), and diabetes (36.4%), which became the main risk factors. The results showed that the implementation of teleconsultation in the HES reduced the possibility of infection and allowed for a more efficient consumption of personal protective equipment.

## 1. Introduction

Since 31 December 2019, when the WHO was alerted about a case of pneumonia of unknown cause in the city of Wuhan, located in China, the life of millions of people around the world suffered a 180-degree turn [1,2]. From this moment on, the propagation of SARS-CoV-2, which causes the COVID-19 disease, experienced an exponential growth, first extending rapidly to the rest of the Chinese provinces, and afterward jumping to the rest of the continents.

COVID-19 propagates very fast [3]. A person can be infected with SARS-CoV-2 due to contact with an infected person [2,4,5], although most of the time, the transmission of the disease is provoked by asymptomatic patients. Nevertheless, it can also be spread by symptomatic patients or those who are in the period of incubation. The disease is fundamentally propagated through droplets that are ejected when an infected person coughs or exhales. This is why it is important to maintain a safe distance (1.5 m) with other people [6,7,8,9].

Millions of people have been infected by SARS-CoV-2 in the world, and more than 646,000 have died [10]. Europe has been especially affected by this pandemic, with at least 3 million cases confirmed up to date, with Russia, the UK, Spain, Italy, Turkey, and Germany being the most affected countries [10,11,12,13].

Since the news of the first case in Spain broke at the end of February 2020, the increase in the number of people diagnosed (280,000) and deceased (28,000) has not stopped rising [12,14]. It is evident that the data are discouraging, but even more so, if we observe that almost 24% of the diagnosed cases have been healthcare personnel [15]. Among the main reasons for infection, we find the absence of personal protection equipment (PPE) at the beginning of the pandemic, due to the worldwide shortage of this equipment.

The hospital emergency service (HES) is one of the main pillars in the care of patients with COVID-19 [16], and structural and processing changes had to be made to provide care for patients with respiratory problems [2]. For this purpose, a new protocol of action was implemented through the use of teleconsultation for patients with respiratory symptoms at the HES from the Hospital de Poniente (Almeria, Spain), which implied the re-organization at the level of care as well as the distribution of patients and consultation rooms.

The objective of the study was to evaluate the effectiveness of a teleconsultation protocol with patients who had been addressed with respiratory symptoms, in the reduction of the consumption of PPE in a HES during the COVID-19 pandemic. The secondary objectives were: (1) to describe the demographic and clinical characteristics, and epidemiological antecedents of patients diagnosed with COVID-19 in the HES, and (2) to estimate the PPE savings that were directly derived from care through teleconsultation.

## 2. Materials and Methods

This is a descriptive and retrospective study, in which data were selected between 18 March and 30 April 2020. Four days after the declaration of the State of Alarm in Spain (14 March 2020), a new teleconsultation protocol was implemented to tend the patients with respiratory difficulties and/or compatible with the disease provoked by COVID-19, at the HES from the Hospital de Poniente (El Ejido, Almeria, Spain), located in a town with a population of 265,000 inhabitants.

### 2.1. Participants

The inclusion criteria for access to the teleconsultation service were: (1) over 14 years old, (2) patients who had a symptomology compatible with acute respiratory problems (cough, fever, and dyspnea), or (3) symptoms compatible with COVID-19. The exclusion criteria were: (1) limited function, (2) language barrier, and (3) a severe situation. In these cases, health care was provided directly in-person.

### 2.2. Outcome Measures

The variables collected to evaluate the telemedicine program were: number of patients attended at the HES during the period of study, number of patients that attended in the respiratory circuit, number of patients who attended through teleconsultation, number of patients who needed in-person attention, number of patients who were discharged through teleconsultation, and patients who returned to the HES and were admitted due to new complications.

Also, other variables were collected, such as: demographic data (sex and age), clinical data (symptoms and diseases and risk factors), and clinical outcomes for COVID19 cases (hospitalization, mechanical ventilation, close contact with probable or confirmed COVID-19 cases, contact with persons with acute respiratory symptoms and home discharge) of the patients who were diagnosed with COVID-19.

To estimate the savings in personal protective equipment (PPE), only the number of patients attended through the teleconsultation with a posterior home discharge, who did not require a hospital admission after a posterior consultation, were taken into account. To estimate this total, the cost of all the equipment components that should have been used by the doctor and the nurse during an in-person consultation were added. In addition, the disinfection and cleaning of the ER and teleconsultation cabins were assessed. The prices were provided by the management unit from the hospital.

### 2.3. Intervention

The HES installations were adapted to facilitate the care protocol of patients with respiratory problems, where the resources necessary for the teleconsultation were installed: four computer systems (two for the doctor’ stations and two for the patient’s cabins) a webcam-based communication system, speakers in the patient’s cabin and headphones with microphones in the doctor’s cabin. The care equipment included in the teleconsultation cabin were a portable blood pressure monitor with a pulse oximeter and screen, a thermometer, and a digital stethoscope with WiFi connection.

**Figure 1:**
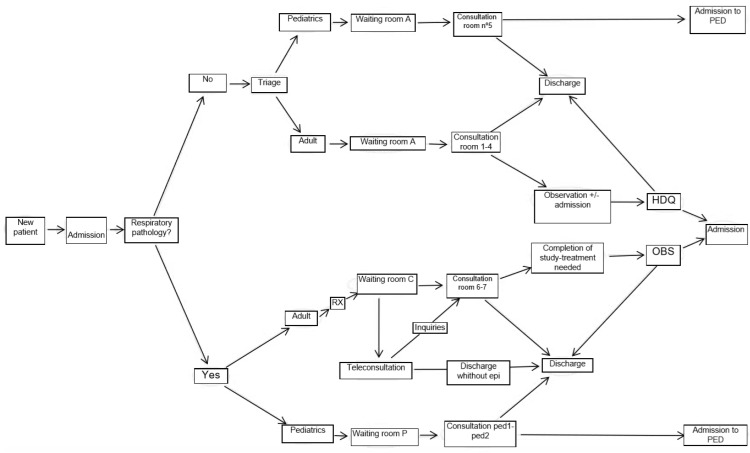
Respiratory Circuit.

The implementation of the teleconsultation was performed by integrating it as another piece of the “Respiratory Patient” circuit of the HES. The main measures of the implemented protocol are summarized in the following steps (Figure 1):

Step 0. When entering the emergency services, the patients and the companion were offered a mask, and during admission, the patient was classified by a doctor into two differentiated circuits: (1) non-respiratory and (2) respiratory. During the classification, if COVID-19 was suspected, a set of gloves and a disposable surgical gown were provided for their use during their hospital stay.

Step 1. Respiratory circuit: All the patients that showed symptoms that seemed to be compatible with COVID-19 were included (fever, cough, atony, etc.).

Step 2. The different circuits that were to be followed were explained to the patient. To minimize posterior movements, a custodian accompanied the patient to the Radiology Unit for radiography of the thorax. Afterward, the patient was taken to a waiting room that was different for patients included in the “non-respiratory circuit”. This waiting room provided access to the telemedicine consultation cabins. Within each cabin, printers were placed (for sending information), as well as informational sheets with telephones and recommendations of interest, and explanatory posters with infographic about the basic use of the instruments, which the patients would have to use later.

Step 3. Consulting of patients with respiratory problems. The ER Physician who attended the patient in this first teleconsultation session was in a room next to the cabin, connected to the videoconference circuit and with another device for receiving the stethoscope signal. Also, the doctor had access to the clinical history electronic platform utilized in the Public Health System of Andalusia, named “Ariadna”, which allowed visualizing and editing the clinical history of the patient. Afterward, the patient was called with the normal public address system, asking them to go to a selected cabin. Once inside, the anamnesis started to be created, and the clinical situation of the patient was confirmed. For this: (1) they were asked to wash their hands with a water-alcohol solution, (2) to obtain their temperature, they were instructed on the placement of the digital thermometer, (3) they were shown how to use the pulse oximeter in order to assess their heart rate and oxygen saturation, (4) if needed, the blood pressure was also taken, (5) the patient was asked to turn on the digital stethoscope, and the proper procedure for this was explained to them.

With all of this information, the doctor in charge had the basic vital signs available, as well as the auscultation parameters and radiological study with the thorax radiography. In the case that the case was “slight” or non-susceptible to additional measures, the doctor prescribed a treatment and issued a discharge report through the printer located in the patient’s cabin. The outpatient clinical monitoring, if complications were inexistent, would be performed by the primary care physician. In the case of detecting a symptom or sign of alarm during the teleconsultation, the patient was taken to the next door consultation room for in-person care and to complete the study that could be needed (by the care team who was already protected with the corresponding PPEs), after which the patient was taken to the observation room or another specific area if needed.

After the consultation, the nursing auxiliary care technician designated to the consultation disinfected the equipment utilized previously and the cleaning personnel disinfected the common areas utilized in the waiting room and cabin.

### 2.4. Ethical Considerations

The study was approved by the Regional Ethics Committee for Health Research (CEIC-AL: 71/2020). The implementation of the protocol was conducted following the guidelines of ethics and research set by the Declaration of Helsinki [17]. All the authors confirm that all the measures necessary were adopted to guarantee the privacy of the identifying data of the patients and the information collected. Also, as the personal data is confidential, they were treated according to the Organic Law on the Protection of Personal Data [18].

### 2.5. Statistical Analysis

The statistical analysis was conducted with SPSS software version 25.0 (IBM-SPSS-Inc., Chicago, IL, USA). The categorical variables were summarized through absolute frequencies and percentages, and the chi-square test was utilized for the group comparisons. The continuous variables were described with the median and the interquartile range, and the comparison between groups was performed through Mann–Whitney’s U-test. Differences were considered to be statistically significant at *p* < 0.05.

## 3. Results

**Figure 2:**
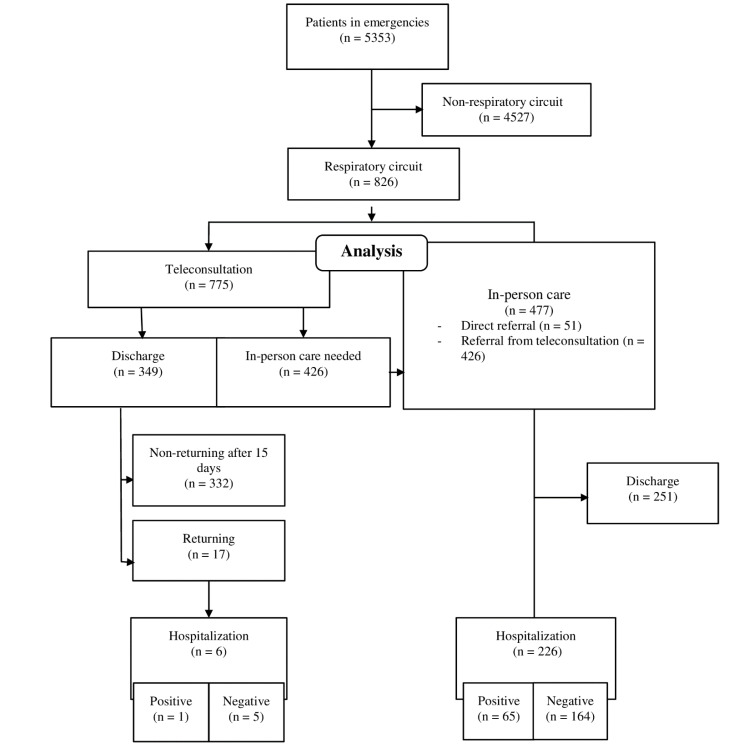
Flow diagram of patients attended in the Respiratory Circuit.

During the study period, 5353 patients attended the HES from the Hospital de Poniente. Of these, 15.4% (*n* = 826) were referred to the Respiratory Circuit. Of these 826 patients, 93.8% (*n* = 775) passed through the teleconsultation, and 42.2% (*n* = 349) of these patients were directly discharged afterwards. The rest of the patients, 57.7% (*n* = 477) needed to be attended in person, and of these, 226 patients were hospitalized, which entails 4.2% of the total number of patients attended in Emergencies (Figure 2).

Throughout the study period, 4.9% (*n* = 17) of the patients discharged from the teleconsultation returned to the HES, and 1.7% (*n* = 6) required hospital admission in the latter consultation. The rest of the patients (98.3%) were attended in an outpatient setting at their corresponding primary care centers.

### 3.1. Demographic, Clinical, and Epidemiological Characteristics of the Patients Diagnosed with the COVID-19 Disease

Among the characteristics shown by the patients attended in the respiratory circuit, it is interesting that 66 were diagnosed with COVID-19. Of these, 57.6% were men, and the mean age was 71 years old (women = 72 vs. men = 71). The most frequent symptoms mentioned by the patients were: cough (77.3%), fever or recent history of fever (77.3%), dyspnea (60.6%), and shivers (25.8%). Also, 89.4% were diagnosed with pneumonia, 25.8% acute renal failure, and 24.2% acute respiratory distress syndrome. The men had a greater prevalence of cough and fever (Table 1).

Of the patients who were diagnosed with COVID-19, 100% of them were hospitalized, and 16.7% needed mechanical ventilation in the ICU. As for their comorbidities, 56.1% of the patients (women = 46.4% vs. men = 63.2%; *p* = 0.176) had one or more diseases and/or risk factors, underlining high blood pressure in 53% (women = 53.6% vs. men 52.6%; *p* = 0.940) and diabetes in 36.4% (women = 35.7% vs. men = 36.8%; *p* = 0.925) of the patients.

During the study period, 18 March and 30 April 2020, all the information related to the registration of the data of the discharged patients (about the second consultation) and the hospitalized patients (about the clinical outcome) was collected.

### 3.2. Savings Derived from Non-Use of the Personal Protective Equipment

Care through teleconsultation avoided the conventional care of 332 patients. The cost of the PPEs per patient and health professionals in the in-person consultation was estimated at 11.27 €. As two health professionals entered the in-person consultation, savings of 7484.61 € in PPEs were estimated.

### 3.3. Disinfection and Cleaning Costs

During the study period, 18 March to 30 April 2020, the disinfection and cleaning of the ER rooms (including teleconsultation cabins) costs were estimated as 41,614.12 €. This supposes an increase of 234.02% in relation to the same period of the year 2019.

## 4. Discussion

During the study period, 5353 patients attended the Hospital Emergency Services at the Hospital de Poniente. The objective of the present study was to evaluate the effectiveness of a teleconsultation protocol with patients who had been addressed with respiratory symptoms, on the reduction of the consumption of personal protective equipment (PPE) in a HES during the COVID-19 pandemic. Telemedicine was developed as a strategy for patient monitoring before their arrival at the HES [19,20,21]. However, reports that describe experiences in its use are lacking, although it was established as a mechanism of care in the HES itself. The closest experience to our use was the application of a telematics triage system for individuals with multiple sclerosis, especially those who were taking immunosuppression medication during the COVID-19 pandemic [22].

In the present study, 15.4% (*n* = 826) of the patients attended were referred to the respiratory circuit. Of these, 93.8% (*n* = 775) were attended through teleconsultation, and 349 were discharged to their homes afterward. This resulted in important savings in the use of PPEs, optimizing a very scarce resource in the beginning stages of the pandemic.

Of the 826 patients attended in the respiratory circuit, 66 were diagnosed with COVID-19, with the most frequent related diagnostic being pneumonia (89.4%). Cough (77.3%), fever, or a recent history of fever (77.3%) were the most frequent symptoms. An aspect that should be highlighted is that 4.9% of the patients discharged through teleconsultation returned to the HES, and 1.7% needed to be hospitalized a few days after. The number of patients addressed with respiratory symptoms during the COVID-19 pandemic has totaled 15.43% of the total care provided at the HES from the Hospital de Poniente. The situation of uncertainty provoked by the risk of infection (of professionals as well as patients), and the high degree of propagation of the infection among the health professionals collective (24% of the total positive cases) [15], has demanded the search for alternative solutions for common practices that are safe and agile.

The differentiation of two care circuits, between patients with respiratory problems and the rest, has been essential at every health care stage. In the specific case of the HES from the Hospital de Poniente, it was necessary to streamline the making of decisions related to these patients, minimizing the exposure of the health professionals. The implementation of a system of teleconsultation based on a system of videoconference and examination elements that were manageable in the distance or with minimum collaboration from the patient, allowed, on the one hand, to stratify the care of most of these patients, to finally decide if in-person care was needed or not. On the other hand, it allowed the doctors to discharge a large number of patients, without the need for direct contact or the use of PPEs (45% of the cases resolved). More than 98% of the cases managed and discharged with this system were able to be monitored without the need for a second examination, thus defining it as effective healthcare that does not result in iatrogenesis.

The clinical and sociodemographic profile of the patients attended through teleconsultation who were positive to COVID-19, shows the existence of a disparity related to the sex of the patients as related to the official statistics. While 57.6% of the patients in this study were men, the report published by the National Network of Epidemiology Surveillance (RENAVE) [23] stated that most were women (56.6%). The age of the patients was also greater (71 vs. 60). And, the percentage of patients who had symptoms was different from those included in the RENAVE report [23], for the symptoms throat pain (13.6 vs. 22%), dyspnea (60.6 vs. 47.6%), diarrhea (15.2 vs. 26.80%), and acute kidney failure (25.8 vs. 5.2%). The percentage of patients who had one or more of the diseases and the risk factors were also lower in the study conducted at the Hospital de Poniente (56.1 vs. 65%).

The inclusion of teleconsultation in the HES has shown to be effective for avoiding exposure in the first stage of care, and for sifting through the respiratory problems in a safe and effective manner with those patients who had slight respiratory symptoms.

This study had diverse limitations. Firstly, the data were subjected to selection bias, as those patients who were clinically more severe, or had some degree of complication, were taken to an in-person consultation room for their assessment and posterior admission if necessary. Secondly, the base situation (dependent patients or with limited comprehension or collaboration) resulted in their having to attend to in-person. Thirdly, the language barrier resulted in the limited comprehension of orders through the video consultation. Besides, it should be noted that the presented validation results are very limited and are not compared with other results yielded by similar research efforts. Also, no technical details concerning the operational and structural characteristics of the employed telemedicine system are demonstrated.

As for the strength of the study, it is clear that this type of system implemented in the HES could be useful for sifting through patients addressed with respiratory symptoms in a safe manner, and could also be efficient for the care of patients with a light pathology who do not require major interventions once the case is evaluated, without exposure of the HES personnel. Besides, the paper provides important information on how to use telemedicine to treat the COVID-19 pandemic. It can actually help hospitals to deal with the critical consequences of the pandemic, such as infecting hospital staff.

In a time in which the necessary resources have been limited (especially related to the PPEs), this type of measure has also facilitated a greater efficiency and savings in their use, as they were utilized only when truly necessary.

In conclusion, the results from the study show that the implementation of Teleconsultation in a HES has served as an effective and efficient measure for the stratification of patients with respiratory symptoms and as a resolute measure for less complex patients. The possibility of patient-doctor spread has been reduced, the use of the PPEs has been optimized, and the making of decisions has been streamlined in the first stage of care of suspected COVID-19 patients.

## 5. Conclusions

The results from the study show that the implementation of teleconsultation in a HES has served as an effective and efficient measure for the stratification of patients with a respiratory pathology and as a resolute measure for less complex patients. The possibility of patient-doctor spread has been reduced, the use of the PPEs has been optimized, and the making of decisions has been streamlined in the first stage of care of suspected COVID-19 patients.

## Figures and Tables

**Table 1:** Demographic and clinical characteristics and epidemiological antecedents of risk. COVID-19 cases at the Hospital Emergency Services at the Hospital of Poniente.

Characteristics		Total	Women	Men	*p*-Value
		N (%)	N (%)	N (%)	
Sex		66 (100%)	28 (42.4%)	38 (57.6%)	
Age	Media (IQR)	71 (62–77)	72 (64–80)	71 (61–76)	0.417
					
Age group (years)	<20	0 (0%)	0 (0%)	0 (0%)	
	20–29	1 (1.5%)	1 (3.6%)	0 (0.0%)	
	30–39	3 (4.5%)	0 (0.0%)	3 (7.9%)	
	40–49	5 (7.6%)	1 (3.6%)	4 (10.5%)	0.350
	50–59	2 (3%)	1 (3.6%)	1 (2.6%)	
	60–69	19 (28.8%)	10 (35.7%)	9 (23.7%)	
	70–79	23 (34.8%)	8 (28.6%)	15 (39.5%)	
	≥80	13 (19.7%)	7 (25%)	6 (15.8%)	
Symptoms	Fever or recent history of fever	51 (77.3%)	21 (75%)	30 (78.9%)	0.705
	Cough	51 (77.3%)	22 (78.6%)	29 (76.3%)	0.829
	Throat pain	9 (13.6%)	5 (17.9%)	4 (10.5%)	0.391
	Dyspnea	40 (60.6%)	17 (60.7%)	23 (60.5%)	0.988
	Shivers	17 (25.8%)	3 (10.7%)	14 (36.8%)	0.016
	Vomiting	7 (10.6%)	3 (10.7%)	4 (10.5%)	0.980
	Diarrhea	10 (15.2%)	5 (17.9%)	5 (13.2%)	0.599
Diagnostics	Pneumonia (radiological or clinical)	59 (89.4%)	25 (89.3%)	34 (89.5%)	0.980
	Acute respiratory distress syndrome	16 (24.2%)	3 (10.7%)	13 (34.2%)	0.028
	Other respiratory diagnostics	10 (15.2%)	1 (3.6%)	9 (23.7%)	0.024
	Acute kidney failure	17 (25.8%)	2 (7.1%)	15 (39.5%)	0.003
Illnesses and risk factors	One or more	37 (56.1%)	13 (46.4%)	24 (63.2%)	0.176
	Cardiovascular disease	29 (43.9%)	9 (32.1%)	20 (52.6%)	0.097
	Respiratory disease	17 (25.8%)	7 (25.0%)	10 (26.3%)	0.904
	Diabetes	24 (36.4%)	10 (35.7%)	14 (36.8%)	0.925
	High blood pressure	35 (53%)	15 (53.6%)	20 (52.6%)	0.940
Hospitalization		66 (100%)	28 (100%)	38 (100%)	-
Mechanical ventilation		11 (16.7%)	1 (3.6%)	10 (26.3%)	0.014
Close contact with probable or confirmed COVID-19 cases		30 (45.5%)	17 (60.7%)	13 (34.2%)	0.033
Contact with person with acute respiratory disease		22 (33.3%)	10 (35.7%)	12 (31.6%)	0.725
Discharged home		1 (1.5%)	1 (3.6%)	0 (0%)	0.240

IQR, interquartile range; ICU, intensive care unit.
